# Supplementary material for: A Likelihood Approach for Real-Time Calibration of Stochastic Compartmental Epidemic Models
Source: PLoS Comput Biol. 2017 Jan 17;13(1):e1005257. doi: 10.1371/journal.pcbi.1005257 (PMC5240920; doi:10.1371/journal.pcbi.1005257)
Supplement: S1 File — (TAR.GZ) [file pcbi.1005257.s014.tar.gz › HSPH_Online-SI-Revision/EnKF07/n100-extreme/n100-extreme_EnKF2_table0.pdf]

1	newly 3 weeks spec	7253.	1767.89	{133., 3472.}	84.4954	1.00133	newly 3 weeks cumul	27162.	11846.8	{807., 20780.}	88.3232	0.809015	newly 5 weeks spec	4336.	447.21	{28., 1177.}	90.3231	1.36077	newly 5 weeks cumul	37280.	13150.7	{911., 22660.}	85.9638	0.869914	newly 54 weeks cumul	48774.	13809.2	{946., 23024.}	85.0547	0.942478
2	newly 3 weeks spec	5965.	2354.04	{109., 4632.}	91.7237	1.0162	newly 3 weeks cumul	20994.	5602.86	{505., 16058.}	81.2257	0.955914	newly 5 weeks spec	3796.	2095.7	{36., 8766.}	97.3767	1.05031	newly 5 weeks cumul	29744.	10176.5	{603., 32373.}	82.5297	0.941206	newly 50 weeks cumul	42279.	22281.2	{760., 82588.}	83.233	0.894672
3	newly 3 weeks spec	6513.	708.28	{0., 1145.}	95.3069	2.21812	newly 3 weeks cumul	27002.	12980.7	{493., 31839.}	91.2555	0.871576	newly 5 weeks spec	3181.	400.67	{0., 673.}	95.0207	3.00068	newly 5 weeks cumul	34839.	13889.7	{722., 40925.}	88.0741	0.911666	newly 55 weeks cumul	41209.	15612.9	{792., 40925.}	84.5106	0.897033
4	newly 3 weeks spec	6774.	404.86	{12., 472.}	96.3484	1.85193	newly 3 weeks cumul	33063.	2520.02	{424., 2349.}	92.6632	1.39624	newly 5 weeks spec	2428.	187.21	{0., 197.}	98.8373	2.40399	newly 5 weeks cumul	39680.	2996.12	{463., 2846.}	93.023	1.43824	newly 59 weeks cumul	42886.	3275.26	{471., 3188.}	93.4488	1.45548
5	newly 3 weeks spec	8431.	2306.11	{102., 9521.}	86.9995	1.1329	newly 3 weeks cumul	31964.	17702.	{1436., 76682.}	93.4348	0.810568	newly 5 weeks spec	4440.	559.71	{9., 681.}	93.0286	1.68498	newly 5 weeks cumul	42439.	19328.	{1529., 80243.}	90.2861	0.874078	newly 56 weeks cumul	51326.	20259.4	{1767., 89688.}	88.1767	0.916988
6	newly 3 weeks spec	7676.	2336.13	{419., 3816.}	82.0786	0.96026	newly 3 weeks cumul	28666.	14017.8	{3944., 30040.}	74.7191	0.596109	newly 5 weeks spec	4357.	610.42	{81., 1086.}	89.4414	1.26473	newly 5 weeks cumul	38952.	15810.8	{4098., 31655.}	76.1488	0.657096	newly 54 weeks cumul	50195.	16608.9	{4131., 31915.}	77.448	0.72487
7	newly 3 weeks spec	7326.	1084.31	{2., 684.}	99.9125	2.05583	newly 3 weeks cumul	27951.	5330.3	{259., 2704.}	98.7963	1.41959	newly 5 weeks spec	3953.	443.36	{0., 411.}	98.0739	2.47085	newly 5 weeks cumul	37123.	6460.4	{317., 3671.}	97.9416	1.48029	newly 52 weeks cumul	46099.	7424.9	{357., 4958.}	96.7525	1.52558
8	newly 3 weeks spec	7809.	2237.72	{442., 3611.}	83.2632	0.909266	newly 3 weeks cumul	28877.	13120.9	{3790., 28480.}	77.2658	0.641731	newly 5 weeks spec	4404.	616.67	{83., 1073.}	89.6124	1.22147	newly 5 weeks cumul	39237.	14907.8	{4056., 29992.}	78.3492	0.70084	newly 54 weeks cumul	50550.	15708.1	{4111., 30145.}	79.1755	0.767249
9	newly 3 weeks spec	8491.	1309.02	{0., 1783.}	89.8549	1.92426	newly 3 weeks cumul	32010.	14045.	{3469., 21662.}	84.5621	0.766235	newly 5 weeks spec	4649.	304.51	{0., 434.}	95.9968	2.19086	newly 5 weeks cumul	43099.	14981.2	{3626., 22496.}	84.2706	0.846787	newly 54 weeks cumul	54004.	15299.6	{3653., 22612.}	84.0715	0.917968
10	newly 3 weeks spec	3970.	2830.79	{393., 3193.}	91.5297	0.547707	newly 3 weeks cumul	22883.	15154.5	{4166., 15771.}	85.751	0.496856	newly 5 weeks spec	1101.	894.78	{42., 1586.}	75.6276	0.611072	newly 5 weeks cumul	26005.	17609.2	{5251., 16531.}	84.3416	0.477389	newly 58 weeks cumul	27241.	19198.2	{6297., 20138.}	80.8315	0.442739
11	newly 3 weeks spec	6532.	1036.3	{5., 1854.}	95.5723	1.76985	newly 3 weeks cumul	23257.	5479.09	{224., 10993.}	93.7347	1.27015	newly 5 weeks spec	3974.	476.51	{0., 519.}	95.6077	2.33423	newly 5 weeks cumul	32341.	6643.05	{294., 12554.}	93.3683	1.33924	newly 52 weeks cumul	43101.	7946.42	{329., 13555.}	92.4486	1.40336
12	newly 3 weeks spec	8024.	373.48	{128., 677.}	95.3455	1.49509	newly 3 weeks cumul	31244.	5280.49	{897., 6770.}	93.2159	1.10451	newly 5 weeks spec	4154.	118.36	{6., 331.}	97.1507	2.0438	newly 5 weeks cumul	41376.	5590.56	{1015., 7712.}	93.1738	1.17366	newly 53 weeks cumul	50863.	5860.5	{1074., 8235.}	93.1706	1.23026
13	newly 3 weeks spec	7496.	1064.65	{32., 678.}	100.158	1.64491	newly 3 weeks cumul	24982.	3253.46	{288., 2406.}	92.1379	1.36827	newly 5 weeks spec	4998.	394.23	{4., 469.}	95.5764	1.99232	newly 5 weeks cumul	36270.	4382.18	{325., 3510.}	93.78	1.44428	newly 51 weeks cumul	54152.	5356.9	{357., 5840.}	92.582	1.52109
14	newly 3 weeks spec	5024.	2573.74	{196., 7868.}	85.3929	0.724672	newly 3 weeks cumul	30056.	20965.6	{3382., 84090.}	86.9906	0.558737	newly 5 weeks spec	1153.	638.28	{6., 667.}	102.413	0.967702	newly 5 weeks cumul	33840.	22804.	{3803., 87456.}	87.0839	0.570163	newly 56 weeks cumul	34818.	23863.6	{4227., 90665.}	87.9605	0.563901
15	newly 3 weeks spec	5483.	2414.93	{193., 10966.}	94.9095	0.968716	newly 3 weeks cumul	26035.	17136.5	{2138., 69002.}	90.2361	0.648682	newly 5 weeks spec	1932.	590.18	{5., 880.}	97.472	1.35761	newly 5 weeks cumul	31301.	18852.2	{2163., 72282.}	90.0276	0.682642	newly 56 weeks cumul	33894.	19752.2	{2878., 95319.}	90.0543	0.692003
16	newly 3 weeks spec	6616.	2443.77	{277., 8538.}	86.0784	0.907842	newly 3 weeks cumul	30832.	19746.9	{2752., 68232.}	85.1781	0.60584	newly 5 weeks spec	2584.	560.72	{9., 995.}	92.0077	1.36001	newly 5 weeks cumul	37486.	21404.5	{3271., 77982.}	84.3392	0.641765	newly 56 weeks cumul	41249.	22244.	{3801., 89223.}	83.8733	0.655448
17	newly 3 weeks spec	6952.	971.24	{34., 738.}	99.7186	1.61943	newly 3 weeks cumul	24739.	3375.14	{310., 4097.}	91.0721	1.34407	newly 5 weeks spec	4470.	369.37	{3., 438.}	96.7488	1.99803	newly 5 weeks cumul	34818.	4422.14	{348., 7187.}	92.8588	1.41982	newly 51 weeks cumul	48456.	5233.96	{402., 8568.}	92.153	1.49381
18	newly 3 weeks spec	7355.	570.95	{8., 810.}	96.5361	1.83121	newly 3 weeks cumul	26994.	2974.2	{212., 3823.}	94.3184	1.39516	newly 5 weeks spec	4020.	320.84	{0., 404.}	95.3796	2.28675	newly 5 weeks cumul	36463.	3704.06	{245., 4864.}	94.1076	1.45011	newly 52 weeks cumul	45100.	4902.35	{263., 5443.}	93.2884	1.47853
19	newly 3 weeks spec	8310.	1427.37	{0., 2853.}	87.925	1.69641	newly 3 weeks cumul	31919.	15581.8	{647., 27090.}	87.3503	0.796129	newly 5 weeks spec	4512.	321.61	{0., 418.}	95.857	2.0109	newly 5 weeks cumul	42585.	16568.6	{647., 28436.}	85.9591	0.866368	newly 54 weeks cumul	52753.	16906.	{780., 28596.}	85.0702	0.928471
20	newly 3 weeks spec	6547.	947.24	{72., 1505.}	89.2076	1.47394	newly 3 weeks cumul	38691.	6063.18	{821., 6706.}	91.1042	1.24664	newly 5 weeks spec	1542.	416.04	{2., 538.}	106.281	1.73472	newly 5 weeks cumul	43499.	7086.01	{896., 8353.}	89.2618	1.24934	newly 59 weeks cumul	44967.	7850.39	{896., 9515.}	89.3022	1.24957
21	newly 3 weeks spec	7119.	2376.83	{165., 9556.}	88.2384	1.04598	newly 3 weeks cumul	29356.	18210.	{1681., 69679.}	92.1921	0.735176	newly 5 weeks spec	3172.	561.28	{7., 785.}	94.1148	1.50895	newly 5 weeks cumul	37313.	19847.4	{2317., 73305.}	90.2043	0.786575	newly 56 weeks cumul	42719.	20743.5	{2801., 92739.}	89.0352	0.814481
22	newly 3 weeks spec	6759.	1984.68	{568., 3143.}	83.5328	0.809626	newly 3 weeks cumul	29716.	10025.8	{2364., 12547.}	89.036	0.805596	newly 5 weeks spec	2827.	833.5	{147., 1366.}	81.0952	0.802419	newly 5 weeks cumul	37047.	12077.	{3160., 16230.}	86.1982	0.786427	newly 57 weeks cumul	41620.	14810.4	{4242., 18615.}	83.0897	0.729646
23	newly 3 weeks spec	6270.	1754.58	{136., 2077.}	95.4571	1.03723	newly 3 weeks cumul	22584.	5022.32	{944., 4751.}	90.6858	0.967764	newly 5 weeks spec	3912.	1168.35	{27., 3796.}	79.8678	1.05204	newly 5 weeks cumul	31596.	7548.43	{1266., 11862.}	88.7532	0.946572	newly 50 weeks cumul	43028.	21052.7	{1487., 85519.}	86.9452	0.836894
24	newly 3 weeks spec	7567.	1140.04	{88., 2051.}	90.5418	1.19623	newly 3 weeks cumul	27322.	3999.86	{397., 7218.}	90.5288	1.12788	newly 5 weeks spec	4888.	968.9	{15., 2891.}	84.2557	1.29738	newly 5 weeks cumul	38440.	5992.77	{533., 11286.}	89.3742	1.11475	newly 50 weeks cumul	52801.	16769.9	{1539., 84686.}	86.1208	1.0188
25	newly 3 weeks spec	6782.	422.71	{78., 807.}	93.7672	1.41067	newly 3 weeks cumul	30207.	5649.58	{799., 8139.}	92.1455	1.05735	newly 5 weeks spec	2743.	151.2	{2., 434.}	94.4878	1.84202	newly 5 weeks cumul	37399.	6036.92	{861., 8156.}	91.8731	1.09371	newly 53 weeks cumul	41988.	6385.62	{875., 8235.}	91.4941	1.10656
26	newly 3 weeks spec	7069.	980.29	{23., 701.}	100.068	1.69823	newly 3 weeks cumul	23874.	3312.4	{181., 2467.}	92.3516	1.39651	newly 5 weeks spec	4701.	382.13	{2., 482.}	96.6175	2.11207	newly 5 weeks cumul	34401.	4396.55	{205., 3703.}	93.2234	1.4754	newly 51 weeks cumul	50936.	5284.61	{208., 7004.}	92.2794	1.57019
27	newly 3 weeks spec	6335.	929.47	{0., 634.}	97.7842	2.22059	newly 3 weeks cumul	22631.	5356.41	{246., 8679.}	94.268	1.25003	newly 5 weeks spec	3937.	402.31	{0., 318.}	97.4592	2.51701	newly 5 weeks cumul	31837.	6367.67	{306., 9780.}	94.478	1.33829	newly 52 weeks cumul	45079.	7175.33	{364., 9908.}	94.2276	1.44611
28	newly 3 weeks spec	7243.	2168.84	{122., 6577.}	92.3559	1.12223	newly 3 weeks cumul	27640.	14536.4	{1516., 27228.}	86.9726	0.721278	newly 5 weeks spec	4186.	531.44	{7., 1077.}	91.72	1.68689	newly 5 weeks cumul	37414.	16084.5	{1724., 31022.}	86.5235	0.796813	newly 56 weeks cumul	48001.	17086.6	{2014., 62936.}	85.635	0.864198
29	newly 3 weeks spec	6769.	1178.13	{185., 1886.}	86.6289	1.03043	newly 3 weeks cumul	23489.	3915.84	{980., 6069.}	85.723	0.957395	newly 5 weeks spec	4398.	1082.07	{31., 3515.}	79.3085	1.08865	newly 5 weeks cumul	33395.	6110.16	{1137., 11682.}	84.5143	0.944333	newly 50 weeks cumul	46315.	18845.9	{1706., 85377.}	82.0498	0.839178
30	newly 3 weeks spec	8192.	390.34	{14., 413.}	96.4934	1.91625	newly 3 weeks cumul	34290.	2172.52	{360., 2457.}	93.6643	1.44295	newly 5 weeks spec	3775.	185.32	{0., 181.}	98.0593	2.55283	newly 5 weeks cumul	43617.	2640.08	{360., 2703.}	94.0989	1.50979	newly 59 weeks cumul	50418.	2930.56	{360., 2920.}	94.5636	1.55533
31	newly 3 weeks spec	7625.	682.12	{12., 778.}	94.3441	1.73712	newly 3 weeks cumul	37142.	5992.27	{244., 11104.}	91.1499	1.30901	newly 5 weeks spec	2494.	185.55	{1., 128.}	97.2466	2.24715	newly 5 weeks cumul	43977.	6524.22	{248., 11410.}	91.033	1.35441	newly 60 weeks cumul	46875.	6785.94	{248., 11449.}	90.7925	1.37261
32	newly 3 weeks spec	6240.	1498.48	{451., 2154.}	85.5853	0.892784	newly 3 weeks cumul	27473.	8015.49	{1956., 9446.}	90.093	0.864037	newly 5 weeks spec	2353.	608.87	{126., 1237.}	78.8122	0.832774	newly 5 weeks cumul	33807.	9515.23	{2638., 11628.}	87.3002	0.841957	newly 57 weeks cumul	37286.	11775.1	{3216., 15918.}	84.4447	0.779697
33	newly 3 weeks spec	8467.	771.87	{0., 1649.}	95.2374	2.4142	newly 3 weeks cumul	28949.	12865.2	{455., 37028.}	87.5443	0.911477	newly 5 weeks spec	5209.	391.72	{0., 1093.}	96.4811	3.32647	newly 5 weeks cumul	40750.	13784.2	{469., 41032.}	84.9503	0.991183	newly 55 weeks cumul	56254.	15098.7	{496., 41032.}	84.4044	1.06051
34	newly 3 weeks spec	6053.	1219.93	{100., 1556.}	95.6982	1.27228	newly 3 weeks cumul	19976.	4005.67	{375., 3200.}	91.9924	1.11564	newly 5 weeks spec	4286.	768.34	{11., 2890.}	86.804	1.42206	newly 5 weeks cumul	29425.	5709.18	{593., 8252.}	91.1195	1.12367	newly 50 weeks cumul	43351.	16779.9	{1435., 83592.}	88.7029	1.00808
35	newly 3 weeks spec	8823.	667.67	{0., 1468.}	95.5737	2.47583	newly 3 weeks cumul	31843.	9242.04	{441., 20515.}	87.0019	1.06255	newly 5 weeks spec	5272.	381.91	{0., 610.}	96.6918	3.38865	newly 5 weeks cumul	44053.	10119.2	{465., 20527.}	86.9609	1.14516	newly 55 weeks cumul	57367.	11589.4	{506., 20829.}	87.2173	1.19217
36	newly 3 weeks spec	6496.	834.42	{118., 853.}	96.1659	1.29578	newly 3 weeks cumul	23327.	6466.28	{1114., 8649.}	88.8814	0.907656	newly 5 weeks spec	4189.	282.44	{11., 268.}	97.9594	1.87035	newly 5 weeks cumul	32804.	7235.24	{1230., 8692.}	90.3774	1.00503	newly 53 weeks cumul	44915.	7641.41	{1254., 8692.}	91.0454	1.11231
37	newly 3 weeks spec	6929.	2502.71	{435., 3039.}	87.4107	0.776535	newly 3 weeks cumul	31730.	13940.2	{4410., 14083.}	82.398	0.612388	newly 5 weeks spec	2540.	786.51	{19., 1499.}	75.6445	0.923306	newly 5 weeks cumul	38553.	16085.7	{5464., 17103.}	81.8846	0.62022	newly 58 weeks cumul	42395.	17568.	{6342., 19230.}	79.6783	0.602966
38	newly 3 weeks spec	5419.	3187.76	{491., 3725.}	86.8175	0.604015	newly 3 weeks cumul	32641.	18228.2	{6123., 21708.}	80.5352	0.52822	newly 5 weeks spec	1229.	1003.73	{35., 1891.}	77.2099	0.647704	newly 5 weeks cumul	36650.	20985.4	{7450., 29461.}	79.567	0.510859	newly 58 weeks cumul	37768.	22739.1	{8988., 32105.}	77.1577	0.47952
39	newly 3 weeks spec	5970.	806.4	{6., 1048.}	98.2157	1.78683	newly 3 weeks cumul	21972.	3895.38	{205., 3668.}	96.968	1.37793	newly 5 weeks spec	3152.	407.77	{0., 425.}	97.0485	2.21781	newly 5 weeks cumul	29579.	4867.87	{229., 5597.}	96.1417	1.42926	newly 52 weeks cumul	36448.	6146.78	{230., 6429.}	94.6857	1.45356
40	newly 3 weeks spec	6214.	776.04	{94., 1007.}	96.9414	1.36245	newly 3 weeks cumul	22734.	6402.87	{1122., 14243.}	89.277	0.970807	newly 5 weeks spec	3630.	254.97	{9., 166.}	98.8091	1.89537	newly 5 weeks cumul	31216.	7103.22	{1234., 14606.}	90.8015	1.05954	newly 53 weeks cumul	41146.	7463.84	{1269., 14652.}	91.4212	1.15356
41	newly 3 weeks spec	5376.	2001.89	{330., 3296.}	80.5497	0.801362	newly 3 weeks cumul	21206.	13275.3	{3118., 25964.}	82.481	0.550407	newly 5 weeks spec	2830.	445.71	{56., 1088.}	87.7276	1.13998	newly 5 weeks cumul	27896.	14640.9	{3261., 27545.}	79.1402	0.592472	newly 54 weeks cumul	33607.	15215.9	{3274., 27728.}	78.1596	0.635107
42	newly 3 weeks spec	4668.	2430.26	{345., 2670.}	91.2768	0.679708	newly 3 weeks cumul	24350.	13394.6	{3927., 15288.}	85.2065	0.567791	newly 5 weeks spec	1451.	764.18	{16., 1382.}	74.5637	0.78338	newly 5 weeks cumul	28470.	15504.3	{5058., 15421.}	84.2754	0.559004	newly 58 weeks cumul	30111.	16938.2	{5739., 17329.}	81.5872	0.526694
43	newly 3 weeks spec	8069.	1490.26	{65., 1199.}	99.8637	1.48017	newly 3 weeks cumul	29202.	5157.87	{520., 4787.}	94.9958	1.27626	newly 5 weeks spec	4750.	417.35	{8., 694.}	92.6663	1.71508	newly 5 weeks cumul	40208.	6411.56	{626., 7095.}	94.5593	1.32541	newly 51 weeks cumul	53458.	7410.86	{688., 7675.}	92.2435	1.36684
44	newly 3 weeks spec	6354.	3444.51	{715., 11993.}	92.4751	0.680043	newly 3 weeks cumul	27932.	14297.1	{4098., 43651.}	84.8777	0.618291	newly 5 weeks spec	2650.	1508.59	{142., 1600.}	94.9921	0.721221	newly 5 weeks cumul	34711.	18139.6	{5008., 75773.}	86.2222	0.624485	newly 57 weeks cumul	39102.	21124.6	{5405., 92140.}	84.3598	0.601879
45	newly 3 weeks spec	4797.	2540.85	{285., 11609.}	93.72	0.813362	newly 3 weeks cumul	26104.	18797.3	{2739., 66639.}	83.49	0.554672	newly 5 weeks spec	1420.	596.58	{13., 1069.}	96.2465	1.10812	newly 5 weeks cumul	30202.	20560.1	{3306., 79480.}	84.0742	0.574807	newly 56 weeks cumul	31840.	21425.3	{4005., 87415.}	84.3193	0.574641
46	newly 3 weeks spec	8356.	2226.63	{241., 6990.}	85.7423	1.02188	newly 3 weeks cumul	33903.	18160.6	{2047., 58502.}	88.6492	0.716089	newly 5 weeks spec	3539.	532.45	{17., 631.}	94.0862	1.46199	newly 5 weeks cumul	42756.	19698.	{2542., 60780.}	87.164	0.762975	newly 56 weeks cumul	48435.	20647.4	{2968., 78282.}	86.4239	0.780983
47	newly 3 weeks spec	6682.	2349.05	{331., 2618.}	89.7999	0.826712	newly 3 weeks cumul	30081.	13172.5	{4029., 13711.}	84.1809	0.63513	newly 5 weeks spec	2681.	739.5	{9., 1393.}	79.9791	1.09526	newly 5 weeks cumul	37114.	15212.4	{4899., 14621.}	83.8678	0.649845	newly 58 weeks cumul	40818.	16631.7	{5975., 17709.}	81.7373	0.632703
48	newly 3 weeks spec	8702.	2841.87	{414., 3092.}	86.4779	0.831773	newly 3 weeks cumul	37680.	16287.2	{4943., 20981.}	83.7654	0.64534	newly 5 weeks spec	3585.	916.58	{25., 1760.}	80.0954	1.05486	newly 5 weeks cumul	46811.	18771.4	{6429., 28297.}	82.5008	0.659722	newly 58 weeks cumul	52174.	20578.9	{7140., 30871.}	80.1754	0.649765
49	newly 3 weeks spec	4127.	653.66	{61., 607.}	95.639	1.38069	newly 3 weeks cumul	27465.	6095.94	{669., 11298.}	90.4106	1.09354	newly 5 weeks spec	921.	158.63	{4., 77.}	100.544	1.70295	newly 5 weeks cumul	30359.	6563.04	{756., 11566.}	90.4781	1.1117	newly 60 weeks cumul	31206.	6747.2	{805., 11598.}	90.5474	1.11688
50	newly 3 weeks spec	6476.	1302.35	{52., 1243.}	101.211	1.44943	newly 3 weeks cumul	23838.	4167.11	{452., 4355.}	91.3726	1.18989	newly 5 weeks spec	3992.	503.15	{5., 896.}	93.3434	1.8028	newly 5 weeks cumul	32964.	5555.67	{485., 8039.}	91.8837	1.24356	newly 51 weeks cumul	44879.	7791.16	{498., 11885.}	87.8117	1.27096
